# Structural Basis of the Novel *S. pneumoniae* Virulence Factor, GHIP, a Glycosyl Hydrolase 25 Participating in Host-Cell Invasion

**DOI:** 10.1371/journal.pone.0068647

**Published:** 2013-07-16

**Authors:** Siqiang Niu, Miao Luo, Jian Tang, Hua Zhou, Yangli Zhang, Xun Min, Xuefei Cai, Wenlu Zhang, Wenchu Xu, Defeng Li, Jingjin Ding, Yonglin Hu, Dacheng Wang, Ailong Huang, Yibin Yin, Deqiang Wang

**Affiliations:** 1 Department of Laboratory Medicine, Chongqing Medical University, Chongqing, People’s Republic of China; 2 Key Laboratory of Molecular Biology on Infectious Disease, Chongqing Medical University, Chongqing, People’s Republic of China; 3 National Laboratory of Biomacromolecules, Institute of Biophysics, Chinese Academy of Sciences, Beijing, People’s Republic of China; 4 The First Affiliated Hospital, Chongqing Medical University, Chongqing, People’s Republic of China; Montana State University, United States of America

## Abstract

Pathogenic bacteria produce a wide variety of virulence factors that are considered to be potential antibiotic targets. In this study, we report the crystal structure of a novel *S. pneumoniae* virulence factor, GHIP, which is a streptococcus-specific glycosyl hydrolase. This novel structure exhibits an α/β-barrel fold that slightly differs from other characterized hydrolases. The GHIP active site, located at the negatively charged groove in the barrel, is very similar to the active site in known peptidoglycan hydrolases. Functionally, GHIP exhibited weak enzymatic activity to hydrolyze the PNP-(GlcNAc)_5_ peptidoglycan by the general acid/base catalytic mechanism. Animal experiments demonstrated a marked attenuation of *S. pneumoniae*-mediated virulence in mice infected by *ΔGHIP*-deficient strains, suggesting that GHIP functions as a novel *S. pneumoniae* virulence factor. Furthermore, GHIP participates in allowing *S. pneumoniae* to colonize the nasopharynx and invade host epithelial cells. Taken together, these findings suggest that GHIP can potentially serve as an antibiotic target to effectively treat streptococcus-mediated infection.

## Introduction

*S. pneumoniae*, commonly known as pneumococcus, is an encapsulated Gram-positive opportunistic human pathogen that frequently colonizes the respiratory epithelium. Although hosts infected with pneumococci remain mostly asymptomatic, infection may cause life-threatening respiratory or even systemic disease, such as bacteremia and peritonitis, depending on the particular strain and host susceptibility [1]. *S. pneumoniae* enters hosts by utilizing various secreted virulence factors to target and manipulate host cells via colonization, adhesion, and invasion, which can result in significant morbidity and mortality [2]. Signature-tagged mutagenesis experiments revealed that numerous genes encoding glycoside hydrolases (GHs) are potential *S. pneumoniae* virulence factors [3].

Glycosyl hydrolases (EC 3.2.1.-) are a widespread group of enzymes that cleave the glycosidic bond in glycoside, glycans, and glycoconjugates. Based on sequence similarities and predicted structures, GHs are classified into 113 families in the Carbohydrate Active enZYmes (CAZy) database [4], [5]. Although these enzymes exhibit common structural folds and active-site topology, they have relatively low sequence similarity with each other and react to a broad range of substrates. The lysozyme subfamily of GHs weakens the stability of bacterial peptidoglycan and facilitates efficient pathogenic bacterial lysis by rapidly cleaving the β-1,4-glycosidic bond between N-acetylmuramic acid (NAM) and N-acetylglucosamine (NAG) [6], [7]. The lysozyme subfamily can be further divided into 5 types: GH22, GH23, GH24, GH25, and GH73. Among them, GH25 enzymes typically exhibit a multi-domain structure, including a catalytic module domain and a choline-binding module domain that is responsible for noncovalently anchoring GH25 to choline moieties on bacterial surfaces [7]. So far, four GH25 enzymes have been identified in *S. pneumoniae* and its bacteriophages, including LytA, LytB, LytC, and Cpl-1; they each contain the typical choline-binding and catalytic modules, and exhibit pneumococcal cell wall lytic enzyme activity. LytA, the main *S. pneumoniae* autolysin, is an N-acetylmuramoyl-l-alanine amidase involved in nasopharyngeal colonization [8], [9]. LytB and LytC, both involved in cell wall biogenesis, inhibit host immune responses, allowing bacteria to establish chronic infection; they also function as virulence factors involved in nasopharyngeal colonization [10]. Cpl-1, encoded by the pneumococcal phage Cp-1, has peptidoglycan hydrolytic activity and causes rapid bacterial lysis in a manner similar to LytA, LytB, and LytC [11], [12]. To date, the three-dimensional structures of 3 GH25 enzymes have been determined, including LytC from *S. pneumoniae* (PDB code 2WW5), Cpl-1 from pneumococcal bacteriophage Cp-1 (PDB code 2J8F), and cellosyl from *Streptomyces coelicolor* (PDB code 1JFX), which is composed of an eight-stranded β-barrel flanked by 7 α helices [6], [11], [13].

Microbial adherence factors are called adhesins. They function at different stages of bacterial infection, such as binding to host-cell receptors or the extracellular matrix. Recently, several *S. pneumonia*–derived pneumococcal adhesins were shown to facilitate adherence to host cells, including enolase, GAPDH, Hic, PavA, Pcho, pneumolysin, PsaA, PspA, and PspC [14], [15]. In addition, some *S. pneumoniae* virulence factors that also facilitate bacterial invasion have been characterized, including hyaluronidase (hylA), neuraminidase (including NanA and NanB), PspA (pneumococcal surface protein A), pneumolysin, and PspC [1], [16]. Some host cell-derived glucoproteins also play important roles in pathogenic bacterial entry, including Factor H (an outer membrane glycoprotein). Agarwal et al. proposed that *S. pneumoniae* invades host cells via a two-step mechanism [17], [18]. Host-derived Factor H initially binds to the PspC adhesin located on the outer membrane of *S. pneumoniae*. Subsequently, the pathogenic bacteria invade host cells by a mechanism regulated by intracellular host signaling molecules, such as phosphatidylinositol 3-kinase. Interestingly, individual virulence factors reduce adhesion and/or invasion do not abolish virulence to host cells, suggesting that virulence factors are involved in *S. pneumoniae* infection [14]. To the detailed infecti mechanism, a future challenge will be characteriz novel virulence factors.

The sp0987 gene in the *S. pneumoniae* TIGR4 strain encodes a putative single-domain protein belonging to the GH25 family. As mutational analysis indicated that this novel protein might be involved in host-cell invasion, we named this protein Glycosyl Hydrolase 25 relating to Invasion Protein (GHIP). To the best of our knowledge, we are one of the first to report that GH25 participates in bacterial host-cell invasion. GHIP shares very low sequence identity (<18%) to other GH25 proteins with known three-dimensional structure, implying that GHIP might exhibit some new structural and/or functional characteristics [19]–[21]. Therefore, in order to better understand the function of this novel virulence factor, we elucidated and now report the X-ray structure of GHIP at 1.86 Å resolution. Functionally, GHIP can hydrolyze peptidoglycan using the active site located at the mouth of its barrel structure; in addition, deleting the *GHIP* gene has a strongly inhibits *S. pneumoniae* virulence.

## Materials and Methods

### Bacterial Strains and Cell Culture

*S. pneumoniae* bacterial strains, including TIGR4, D39, and R6, were cultured on trypticase soy agar plates supplemented with 5% sheep blood (blood agar) or in C+Y (casamino acid & yeast extract) medium. Cultures in the exponential phase of growth were frozen and stored at −80°C in C+Y medium containing 10% glycerol. The A549 (type II epithelial lung carcinoma cells; ATCC) and CNE2 (a nasopharyngeal carcinoma epithelioid *cell line* was provided by Cancer Institute, Sun-Yet Sen University of Medical Sciences Guangzhou, China) were grown in Dulbecco’s modified Eagle’s medium (DMEM) or RPMI 1640 medium, respectively, supplemented with 10% fetal bovine serum (FBS) plus 5 mM glutamine, penicillin G (100 U mL^−1^), and streptomycin (100 µg mL^−1^) at 37°C under 5% CO_2_ [22].

### GHIP Protein Expression and Purification

Purification and crystallization of GHIP from *S. pneumoniae* were carried out as described earlier [23]. The *GHIP* gene fragments were amplified using *S. pneumoniae* TIGR4 genomic DNA as the template. The two PCR products were cloned into the *Eco*R I and *Xho* I sites of pET28a. Protein was expressed in *E. coli* strain BL21 (DE3) grown in LB at 37°C. Once an OD_600_ of 0.4–0.6 was reached, IPTG was added to 0.2 mM, the temperature was lowered to 20°C, and expression was allowed to occur overnight. Cells were then harvested, resuspended in ice-cold buffer A (20 mM Tris–HCl at pH 8.0, 300 mM sodium chloride), and then lysed by sonication. All subsequent purification steps were performed at 4°C. Cell debris was removed by centrifugation at 15,000× *g* for 30 min, and the resulting soluble fraction was applied to an Ni^2+^-NTA affinity resin (Qiagen). After washing (15 mM imidazole in buffer A), the protein was eluted from the resin with 200 mM imidazole in buffer A. The protein was buffer-exchanged in buffer containing 5 mM Tris–HCl pH 8.0 and 10 mM sodium chloride, and concentrated to approximately 50 mg mL^−1^. Selenomethionine-substituted GHIP protein (SeMet-GHIP) was produced in *E. coli* strain B834 (DE3) in synthetic media supplemented with L-selenomethionine and purified using the method described above. The purified GHIP and SeMet-GHIP concentration was adjusted to 20 mg mL^−1^ for crystallization trials.

### Crystallization, Data Collection, and Structure Determination

Crystals of both the native and the SeMet-substituted GHIP proteins were grown using the hanging-drop vapor diffusion method at room temperature. The initial crystallization conditions were screened by the sparse-matrix sampling method using Crystal Screen I and II and PEG/Ion Screen kits (Hampton Research). Ultimately, suitable crystals for diffraction experiments were grown within 10 days at 20°C using vapor diffusion and a reservoir solution containing 26–28% polyethylene glycol (PEG) 3350 and 0.2 M lithium citrate in the presence of 0.1 M Bis-Tris, pH 7.31; the protein concentration was at 10 mg mL^−1^ in 50 mM NaCl and 5 mM Tris-HCl, pH 7.5.

A SAD data set was collected from a single crystal of SeMet-substituted GHIP protein at −173°C on beamline BL17U1 at the Shanghai Synchrotron Radiation Facility (SSRF). Data were collected at one wavelength (λ_peak_ = 0.9793 Å) and processed and scaled to 2.70 Å using the HKL2000 software package [24]. A native data set processed using Mosflm and Scala in the CCP4 suite with 1.86 Å resolution was obtained from a wild-type crystal at 100 K by cryoprotection methods [23], [25].

Both the wild-type and SeMet-substituted crystals are in space group P2_1_2_1_2_1_ with similar cell parameters. Data collection statistics are presented in Table 1. Using the 2.70 Å SAD data and the SOLVE program, the initial phase was calculated [26]. The initial phase was then improved by density modification, and ∼50% of the residues in the model were automatically built using the RESOLVE program [27]. Further model building was manually performed using the WinCoot program, and refinement was performed with Refmac5 in the CCP4 suite [28], [29]. After the model attained a reasonable quality, refinement continued with the 1.86 Å data collected from the native protein crystal. Further cycles of refinement and model building were carried out until the crystallographic R_factor_ and R_free_ converged to 18.0% and 24.5%, respectively. The structural stereochemistry was checked by PROCHECK [30]. Ribbon cartoons and surface representations were prepared using PyMOL [31]. SAD phasing and model refinement statistics are shown in Table 1. The current model was deposited at the Protein Data Bank with PDB code 4FF5.

**Table 1:** X-ray data collection and refinement statistics of *S. pneumoniae* GHIP.

Data set	Native	Peak
Space Group	P2_1_2_1_2_1_	
Cell dimensions (Å)	a = 36.31, b = 40.89, c = 14	6.66
Wavelength (Å)	1.5418	0.9793
Resolution (Å)^a^	50–1.86(1.95–1.86)	40–2.70(2.80–2.70)
Total number of reflections used	76989	146161
Number of unique reflections	17755	6480
Completeness (%)^a^	91.8 (78.2)	99.5 (96.3)
Average Redundancy	4.3 (2.7)	13.0 (12.3)
R_merge_ (%)^ab^	3.3 (11.4)	6.0 (8.2)
Refinement		
R_free_ c	24.50%	
R_factor_ c	18.00%	
Bond length rmsd(Å)^d^	0.009	
Bond angle rmsd(°)^d^	1.361	

a Values in parentheses are for the highest-resolution shells.

b R_merge_ = ∑(I−<I>)/∑I, where I is the observed intensity and <I> is the statistically weighted average intensity of multiple symmetry related observation.

c R_factor_: R = ∑||F_calc_|−|F_obs_|/∑|F_obs_|, where F_calc_ and F_obs_ are the calculated and observed structure factors, respectively. R_free_ is calculated the same as R_factor_ but from a test set containing 5% of data excluded from the refinement calculation.

d rmsd, root-mean-square deviation from ideal geometry.

### Construction of GHIP Mutants

Amino acids Asp56, Asp154, Glu156, and Asp245 were mutated to alanine by site-directed mutagenesis using appropriate primer pairs. The pET28a-GHIP plasmid carrying the *GHIP* gene fragment was used as the template. The mutational plasmids were then used for over-expressing the protein in *E. coli,* and the mutant gene products were purified and processed by methods similar to those performed on the wild-type protein.

### GHIP Enzymatic Activity

GHIP hydrolase activity was examined using N-acetylchitooligosaccharides (PNP-[GlcNAc]_5_)-derived p-Nitrophenyl beta-glycosides as substrates using the methods described by Nanjo et al. with minor changes [32]. The reactions were carried out by mixing the enzyme (3 µM) with the PNP-(GlcNAc)_5_ (0.06 mM) substrate in the 0.1 M sodium acetate (pH 4.0), citrate (pH 5.0–6.0) or sodium phosphate (pH 6.5–9.0) buffers. The mixtures were then incubated at 37°C for 40 h. An equal volume of 0.1 M sodium carbonate was then added to the mixture to terminate the enzymatic reaction. The resulting concentration of the product formed during the reaction was determined by measuring the absorbance of released PNP-(NAG)_5_ at 405 nm.

### Cellular Localization of the GHIP

The cellular localization of GHIP was examined using a fluorescence reporter system by constructing both C- and N-terminal fusions to a fast folding variant of GFP [33]. To construct plasmid pAE03-GHIP plasmid (GHIP-GFP), the DNA fragments containing *GHIP* gene were digested with *Eco*R I and *Nhe* I and inserted into vector pAE03 which had been digested with the same restriction enzymes. Similarly, pJWV25 and the GHIP PCR-amplified genes were digested with *Spe* I and *Not* I and ligated to vector pJWV25 to construct plasmid pJWV25-GHIP (GFP-GHIP). Inserts and flanking regions on all plasmids constructed were confirmed by sequencing. pAE03-GHIP and pJWV25-GHIP derivative strains were obtained by adding plasmids to TIGR4 competent cells of *S. pneumoniae* grown on TSB blood agar plates containing the appropriate antibiotic [33]. Recombination strains were grown at 30°C in C+Y medium without shaking but with an approximate 50% air volume to allow for proper GFP folding. Where relevant, media were supplemented with 0.15 mM ZnCl_2_. For GFP, the excitation light of a 100 W Hg-vapor lamp was limited to 480–500 nm and the emission wavelengths were 509–547 nm (filters from Chroma). Microscopy images were captured using softWoRx (Applied precision).

### Construction of R6*ΔGHIP* and D39*ΔGHIP* Mutants

Constructing the insertion/deletion mutants of *S. pneumoniae GHIP (ΔGHIP*::*ermB*) is a well established method using the long flanking homology polymerase reaction [34]. The ermB cassette was amplified from *S. pneumoniae* CPM8 DNA by PCR. Upstream and downstream fragments were amplified using their specific PCR primers. Overlap extension was performed to generate the Up-Erm-Dw fusion fragment. The resulting fragment was used to transform both *S. pneumoniae* R6 and D39. Briefly, pneumococcal strains D39 and R6 were exposed to the DNA for 90 min at 37°C after treatment with a competence-stimulating peptide. The ensuing culture was plated on blood agar plates containing 0.25 µg mL^−1^ erythromycin. Erm insertions at the loci of *GHIP* were confirmed by DNA sequencing using the PCR products from genomic sequences harvested from *ΔGHIP S. pneumoniae* colonies.

### Evaluating GHIP Virulence

Experiments testing the virulence function of GHIP were performed as described by Blue and Mitchell with minor alterations [35]. Female outbred BALB/c mice aged 9–13 weeks and weighing 30–35 g were used for animal studies. All mice were nourished with sterile water and sterile standard pellet food *ad libitum*. The investigation conforms with the Guide for the Care and Use of Laboratory Animals published by the US National Institutes of Health (NIH Publication No. 85–23, revised 1996). All animal work was carried out under appropriate licensing by the Committee on the Ethics of Animal Experimentation at Chongqing Medical University (Reference Number: 2011-0040). All surgery was performed under sodium pentobarbital anesthesia, and all efforts were made to minimize suffering.

Prior to use in mice, strain D39 and its Δ*GHIP*-deficient mutant were grown in C+Y medium until optical density reached a value of 0.5 at 600 nm. Bacterial cultures were pelleted by centrifugation and frozen in aliquots with 10% glycerol at −80°C. Bacterial counts were determined by thawing an aliquot of bacterial stock, serially diluting the bacteria, and plating onto blood agar plates. When required, aliquots were rapidly thawed, harvested by centrifugation, and resuspended in sterile phosphate-buffered saline (PBS).

For intranasal infection, mice inspired a volume of 50 µL containing 1.0 × 10^8^ CFU of either the virulent pneumococcal strain D39 or the isogenic Δ*GHIP* mutant resuspended in sterile PBS into their nares. The inoculum dose was confirmed by plating on blood agar broth as described above [35]. Symptoms were monitored for 400 h post-infection, and mice were culled prior to reaching, or upon reaching, a moribund state. For intraperitoneal challenge, mice were each injected with 1.0 × 10^3^ CFU of bacteria resuspended in sterile PBS. Symptoms were monitored for 100 h post-infection, and mice were culled prior to reaching, or upon reaching, a moribund state.

Experiments testing bacterial colonization in tissues were performed as described by Blue and Kafka with a slight modification [35], [36]. To enumerate bacteria in different organs after intranasal challenge, mice were sacrificed at 12, 24, and 36 h post-infection, and blood samples, nasopharynxes, and lungs were aseptically collected and washed three times with PBS (pH 7.3). Samples were then homogenized in PBS with a tissue homogenizer (model 200, double insulated) (PRO Scientific, Inc., Oxford, CT) on ice and serially diluted in sterile PBS as appropriate onto culture plates, which were then incubated for approximately 16 h at 37°C in an atmosphere of 95% air, 5% CO_2_. Colonies were then counted and averaged between replicates.

### Adhesion and Invasion Assays on A549 and CNE2 Cells

Evaluating the adhesion and invasion ability of *S. pneumoniae* was performed as previously described with a minor modification [37]. A549 and CNE2 cells were inoculated and grown to confluence in 24-well tissue culture plates up to 1 × 10^5^ CFU mL^−1^ and washed three times with PBS. Exponential-phase cultures of *S. pneumoniae* R6 and its isogenic *ΔGHIP* mutant derivatives (10^8^ CFU) were treated with DMEM (for A549 cells) or RPMI 1640 (for CNE2 cells) medium supplemented with 10% FBS. The bacteria were diluted in the appropriate medium (DMEM for A549 and 1640 for CNE2), and host cells were inoculated at a multiplicity of infection (MOI) of 10 bacteria/cell. After an additional 1 h of incubation at 37°C, the culture fluid was removed from each well, and the monolayers were washed three times with PBS. Cells were then detached from the plates with 100 µL of 0.25% trypsin plus 0.02% EDTA and then lysed by adding 400 µL of Triton X-100 (0.025% in H_2_O). For adhesion experiments, appropriate dilutions were plated on blood agar to determine the numbers of viable bacteria. Values represented the average of three wells from at least two separate experiments.

Invasion experiments were performed as previously described with minor changes [38]. Briefly, the R6 wild-type and *ΔGHIP*-deficient strains were grown to 10^8^ CFU, washed, and diluted in medium supplemented with 10% FBS appropriate for the host-cell type such that the MOI was approximately 10 bacteria/host cell, similar to the previously described adhesion assays. Bacterial suspensions were added to mammalian cells for 2 h, and cells were washed. Fresh medium containing 10 µg mL^−1^ penicillin and 200 µg mL^−1^ gentamicin was added to each well in order to kill all extracellular bacteria. After an additional 1 h of incubation, the monolayers were washed three times with PBS. The cells were then detached from the plates using 100 µL of 0.25% trypsin plus 0.02% EDTA and then lysed by adding 400 µL of Triton X-100 (0.025% in H_2_O). The number of viable bacteria released from the cells was assessed after serially diluting the lysates on agar plates. The cellular invasion experiments were performed in triplicate and repeated twice.

## Results

### Overall Structural Description

The GHIP crystal structure was phased by single-wavelength anomalous dispersion at 3.0 Å resolution and refined against the diffraction data acquired from the native GHIP crystal at 1.86 Å with a 24.5% free R-factor and an 18.0% crystallographic R-factor. The final model consisted of 227 amino acids (residues 15–243) and 200 water molecules. Due to conformational disorder, the N-terminal residues (1–14) were not represented in the electron density and therefore not included in the final model. The root mean square deviation (RMSD) from ideal values of bond lengths and bond angles were 0.009 Å and 1.361°, respectively. In the final model, 87.3% of residues have main-chain torsion angles in the most favored region of the Ramachandran diagram, and the remaining 12.7% of residues occupy additional allowed regions. Data collection and refined statistics are summarized in Table 1.

**Figure 1:**
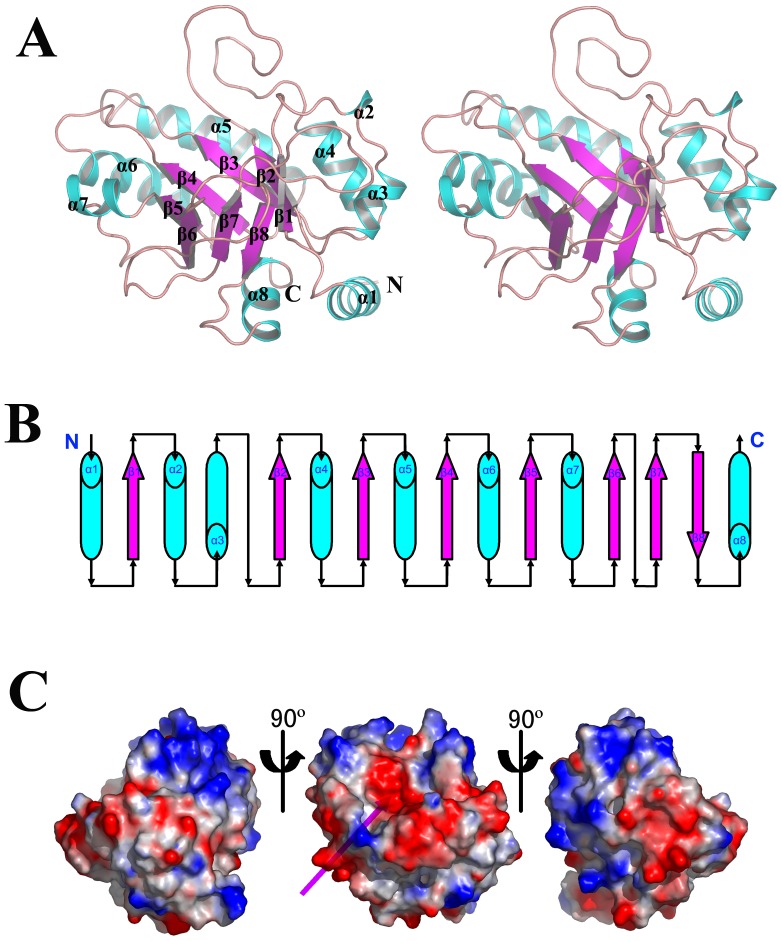
The overall structure of *S. pneumoniae* GHIP. (A) The structure of *S. pneumoniae* GHIP in stereo view. (B) The topological diagram of *S. pneumoniae* GHIP. The barrels and arrows indicate the helix and β-strand structures. (C) The electrostatic potential surface of *S. pneumoniae* GHIP. Saturated red indicates Φ<−10 kiloteslas/e, and saturated blue indicates Φ>10 kiloteslas/e, T = 20°C. The magenta arrow points towards the negatively charged groove.

Only one GHIP molecule was present in the asymmetric unit. The overall structure of GHIP consists of one domain shaped into a flattened ellipsoid with dimensions of 30 Å × 38 Å × 40 Å (Figure 1). The protein exhibits a topoisomerase (TIM) fold that is slightly different from known structures, such as triosephosphate isomerase, glycolate oxidase, isomerases, and hydrolases [39], [40]. GHIP contains an inner ring of seven parallel β-sheets (β1–7) with a protruding β-strand (β8) at the C-terminal region, and an outer wheel composed of eight α-helices surrounds this inner ring (Figure 1). While most of the β-sheets and α-helices exhibit continuous interval distribution, the short 3_10_ (α2) helix connecting β1 and α3 as well as the β6–β8 sheets is connected by a long loop instead of a helix (Figure 1).

**Figure 2:**
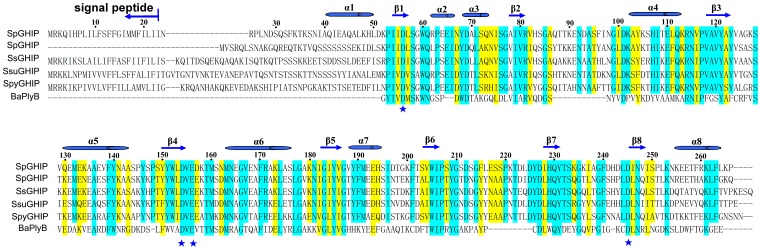
Amino-acid sequence alignment comparing homologs of *S.pneumoniae* GHIP. Sequence alignment of five streptococcus SpGHIP homologs, including SpGHIP from *Streptococcus pneumoniae* TIGR4 (accession number NP_345466), *Streptococcus gordonii* (accession number YP_001450056), *Streptococcus sanguinis* (accession number EGD37983), *Streptococcus pyogenes* (accession number NP_268858), *Streptococcus suis* (accession number EHC03477), and PlyB from the BcpI phage (PDB code 2NW0). Identical and similar residues are shown in *cyan* and *yellow*, respectively. Secondary structure elements of SpGHIP are shown above the sequences. Residues are numbered according to the SpGHIP sequence. Sequence alignment was generated using ClustalW.

The electrostatic potential surface of GHIP displays prominent asymmetry. As shown in Figure 1, 14 amino acids, including Asp56, Ser58, Ser84, Tyr121, Tyr123, Glu154, Glu156, Asp157, Tyr185, Tyr209, Asp212, Ser233, Asp243, and Asp245, form a negatively charged groove at the mouth of the GHIP barrel. Sequence analysis indicates that 11 of these 14 residues are conserved among various bacterial species (Figure 2). Interestingly, the active sites of these characterized enzymes are similarly located at the mouth of TIM barrel structures, suggesting that this negative groove may be a feature of GHIP homologs that relates to their biochemical activity [19], [41].

**Figure 3:**
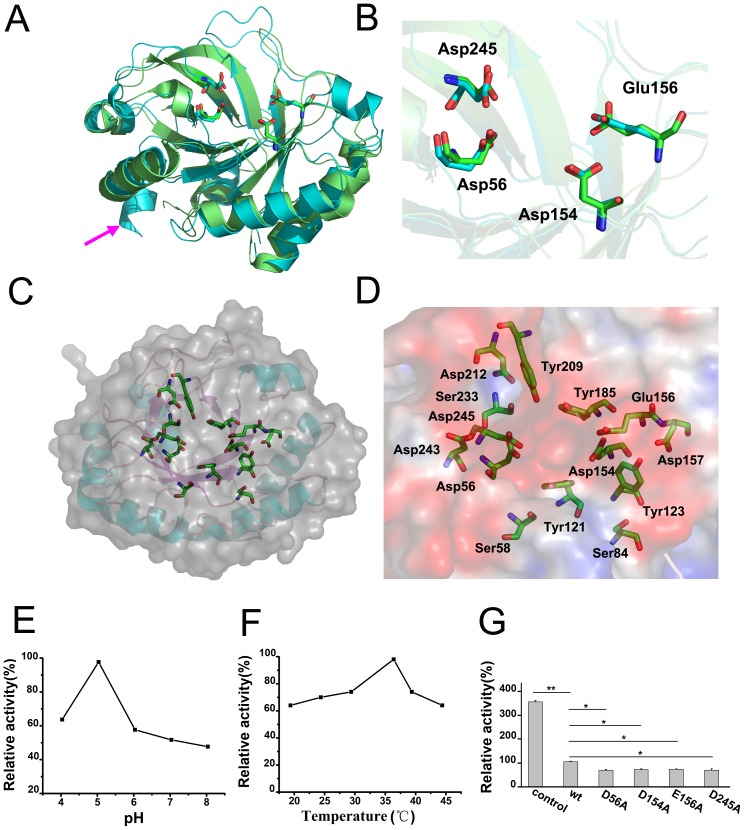
The glycosyl hydrolase activity of *S. pneumoniae* GHIP. (A) *S. pneumoniae* GHIP (green) superimposed on its homolog from *Bacillus anthracis*, PlyB (cyan) (PDB code 2NW0). The magenta arrow points toward the major difference, where the N-terminal regions form a helix (α1) which is absent in PlyB. The sticks represent the four key acidic residues to their enzyme activities, inculding Asp56, Asp154, Glu156, and Asp245 of GHIP and Asp6, Asp90, Asp92, and Asp171 of PlyB. (B) The enlarged image of the four key residues at the active site. Close up view of *S. pneumoniae* GHIP showing overlay of key acidic residues: Asp56, Asp154, Glu156, and Asp245 of GHIP and Asp6, Asp90, Asp92, and Asp171 of PlyB, which exhibit similar locations and orientations. (C) The month of TIM barrel in *S. pneumoniae* GHIP is active site which contains 14 residues in sticks, including Asp56, Ser58, Ser84, Tyr121, Tyr123, Glu154, Glu156, Asp157, Tyr185, Tyr209, Asp212, Ser233, Asp243 and Asp245. The GHIP structure is shown in the context of a transparent (gray) surface. (D) The enlarged image of the 14 residues at the active site and the background is the electrostatic potential surface of *S. pneumoniae* GHIP. Saturated red indicates Φ<−10 kiloteslas/e, and saturated blue indicates Φ>10 kiloteslas/e, T = 20°C. (E) & (F) The temperature and pH activity analyses of *S. pneumoniae* GHIP. Peptidoglycan hydrolase activity was measured at various pH (4.0 to 8.0) and temperatures (25 to 45°C) ranges as described in the Materials and Methods. (G) Hydrolase activity of *S. pneumoniae* GHIP on PNP-(GlcNAc)_5_. Lane 1 represents the positive control using HEWL (egg white lysozyme); lane 2 is native GHIP; lanes 3–6 are active-site mutants D56A, D154A, E156A, and D245A, respectively. Asterisks denote values significantly different from the wild-type strain by Student’s *t*-test (*, P<0.05; **, P<0.01).

BLAST reveals that only streptococcus bacteria encode GHIP homologs, including *Streptococcus pneumoniae, Streptococcus equines, Streptococcus gallolyticus, Streptococcus salivarius,* and *Streptococcus mutans*, suggesting that this novel protein might be species-specific and serve some particular *Streptococcus* function (Figure 2). The GHIP structure was submitted to the DALI server to identify several proteins with similar structural homologies [42]. The top matches belonged to the hydrolase family, including peptidoglycan hydrolases (PlyB) (PDB code 2NW0), endo-N-acetylmuramidases (PDB code 1JFX), and peptidoglycan (PG)_3_ hydrolases (PDB code 2IXU). Among them, PlyB folding is the most similar to GHIP with a RMSD of 1.36 Å (Figure 2B, 3A and 3B), although several structural differences exist between the two similar barrel structures. The major differences are observed at the N-terminal regions (Figure 3A), where the bottom of the barrel formed by α1 (residues 39–49) together with α6 in GHIP is absent in PlyB. In addition, two pronounced differences are apparent at the loop regions (Figure 3A). Whereas loop A in GHIP contains 23 residues (82–104) connecting β2 and α4, the corresponding loop in PlyB is only 8 residues (32–39) long. In addition, GHIP contains an inserted helix (α2) between β1 and α3 in the same place that PlyB contains a shortening loop. Based on the similarities between the two structures, GHIP is predicted to hydrolyze peptidoglycans using an active enzymatic site located at the negatively charged mouth of the TIM barrel, similar to PlyB (Figure 3B, 3C and 3D).

### GHIP Enzyme Activity

GHIP belongs to the GH25 family that can cleave the β1–4 glycosidic bond between NAM and NAG in peptidoglycan. To ascertain if GHIP truly exhibits hydrolase activity, zymography experiments were previously performed [43] and showed that little lysin activity was observed by GHIP on the peptidoglycan in *S. pneumoniae*, *E coli,* or *P. aeruginosa* cell walls. Additionally, *ΔGHIP*-deficient mutant strains did not exhibit any impaired bacterial growth *in vitro*, further supporting that GHIP exhibits little autolysin activity and is therefore not a necessary factor for bacterial survival and growth. When we performed a colorimetric assay evaluating PNP-(GlcNAc)_5_ hydrolysis, however, purified GHIP exhibited peptidoglycan-hydrolyzing activity [32]. Figure 3E shows the pH-activity profiles of PNP-(GlcNAc)_5_ hydrolysis by GHIP over a pH range of 3.0 to 8.0. GHIP exhibited maximum hydrolyzing activity at pH 5.0, and approximately 70% of maximal activity was observed at pH 4.0 or 6.0. The pH-activity profile of PNP-(GlcNAc)_5_ by GHIP was similar to that of hen lysozyme and mouse lysozyme M [44]. The optimum reaction temperature was determined by incubating the assay mixture over a temperature range of 20 to 45°C; the highest peptidoglycan-hydrolyzing activity occurred at 37°C, which decreased by approximately 30% at 30°C and 40°C (Figure 3F). Since GHIP exhibited much lower hydrolytic activity on PNP-(GlcNAc)_5_ than hen egg white lysozyme (HEWL), the synthetic PNP-(GlcNAc)_5_ peptidoglycan may not be a natural GHIP substrate.

To identify the active site of GHIP, ClustalW was used to align multiple sequences, including GHIP and GH25 enzymes from *Streptococcus gordonii*, *Streptococcus sanguinis, Streptococcus pyogenes,* and *Streptococcus suis* [45]. As previously mentioned, GHIP contains a negatively charged groove located at the mouth of its TIM barrel, which is similar to the known TIM enzymes like triosphosphate isomerase and pyruvate kinase (Figure 1C) [46], [47]. Porter et al. proposed that the catalytic machinery comprised four acidic residues equivalent to the Asp6, Asp90, Glu92, and Asp171 in PlyB, which are strictly conserved in the GH25 family [19]. Analogously, the four corresponding residues in GHIP homologs, including Asp56, Asp154, Glu156, and Asp245, are also strictly conserved (Figure 2 and 3B). To further investigate the role of these conserved residues in the hydrolytic function of GHIP, Asp56, Asp154, Glu156, and Asp245 were individually mutated to alanine, and the activity of the four single-point mutants on PNP-(GlcNAc)_5_ were determined by spectroscopy (Figure 3G). All mutants showed lower hydrolytic activity compared to wild-type GHIP, suggesting that these four residues are located at the active site of this novel peptidoglycan hydrolase enzyme. Unfortunately, the complexed GHIP–PNP-(GlcNAc)_5_ structure has not been determined even though the substrate is soaked and co-crystallized, possibly because the crystallization environment may be interfering with substrate incorporation.

### GHIP is a Novel *S. pneumoniae* Virulence Factor

**Figure 4:**
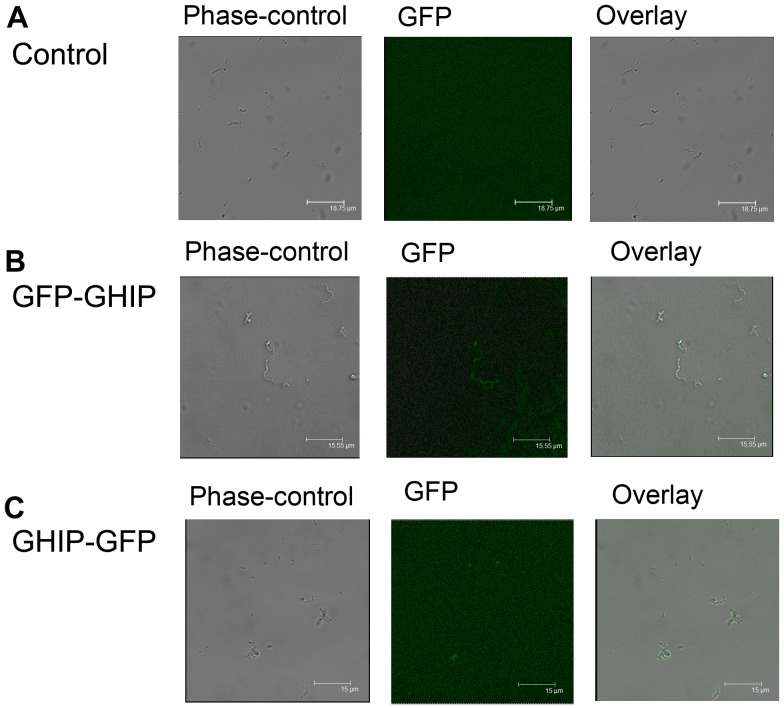
The cellular localization of *S. pneumoniae* GHIP. Cells were grown in C+Y medium in presence of the indicated concentrations of ZnCl_2_ and harvested at mid-exponential growth for observation by automated phase-contrast time-lapse microscopy. (A) The fluorescence microscopy of strain R6 without fusion *GHIP*; (B) The C-terminal GFP fusion (*GHIP-GFP*); (C) The N-terminal GFP fusion GFP (*GFP-GHIP*).

According to Pfam sequence annotation, GHIP contains a predicted N-terminal peptide (residues 1–23), indicating that the protein may normally reside on the surface of *S. pneumonia* [48]. To verify the cellular localization in live *S. pneumoniae,* we constructed GFP fusions to the gene in the *GHIP* locus of TIGR4 strain [33]. Interestingly, both recombinant C-terminal and N-terminal GFP fusions produced fluorescence on the surface of the cell, suggesting that GHIP, similar to the known peptidoglycan hydrolases, is localized to the outer surface of bacteria (Figure 4). As cell surface proteins have historically been implicated as virulence factors in bacterial infectious diseases, GHIP is presumed to also function as an *S. pneumoniae* virulence factor (14). Supporting this, GHIP is not required for cell division and maintenance of cell morphology, as a *ΔGHIP*-deficient mutant created in an encapsulated *S. pneumoniae* D39 strain (using long flanking homology polymerase reaction mutagenesis) did not impair growth or phenotype *in vitro* (C+Y medium) and exhibited a normal cell morphology similar to the wild-type D39 strain.

**Figure 5:**
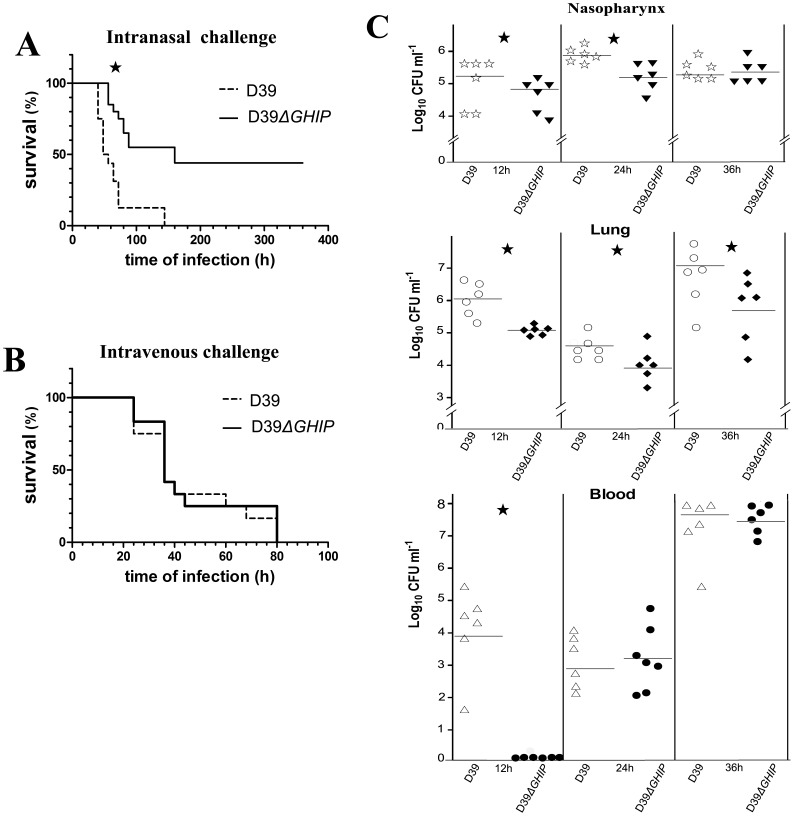
Animal and colonization experiments testing *S. pneumoniae* GHIP function. (A) & (B) The effect of the *GHIP* deletion mutation on virulence. Groups of 18 BALB/c mice were intranasally or intraperitoneally challenged with 1.0 × 10^8^ and 1.0 × 10^3^ CFU, respectively, of D39 or the isogenic *ΔGHIP* mutant. Each datum point represents one mouse. Solid line, wild-type D39; dotted line, *ΔGHIP* mutant. Asterisks denote values significantly different from wild-type by Student’s *t-*test (*, P<0.05). (C) The effect of the *GHIP* deletion mutation on bacteria recovered from nasopharynxes, lungs, and blood samples of BALB/c mice after intranasal challenge. BALB/c mice were intranasally challenged with either wild-type D39 or the *ΔGHIP* mutant at 1.0 × 10^8 ^CFU/mouse. At 12, 24, and 36 h post-infection, six mice from each group were scarified, and the number of recovered bacteria was determined by plating on blood agar. Results are expressed as log_10_ CFUs per gram tissue and represent individually recorded values for each mouse. Horizontal bars represent geometric means. Asterisks denote values significantly different from wild-type by Student’s *t-*test (*, P<0.05).

To study whether GHIP is involved in the bacterial infection process, we compared the ability of wild-type or *ΔGHIP-*mutant D39 strains to infect mice upon challenge by the intranasal or intraperitoneal routes. First, BALB/c mice were intranasally challenged with *S. pneumoniae* D39, and survival was monitored. At 60 h after infection, only 2/18 mice challenged with wild-type D39 survived (death rate 89% and median survival time 60 h); in contrast, 8/10 ten mice challenged with the *ΔGHIP* mutant survived during the entire infection period while 10/18 succumbed to the infection (death rate 56% and median survival time 150 h). This result indicated that the infection progression was much slower in mice infected by the *ΔGHIP* mutant strain than by the wild-type strain (Figure 5A). After intraperitoneal challenge, however, both the wild-type and *ΔGHIP*-deficient D39 strains showed similar survival rates, where the median survival time was 36 h for both groups (Figure 5B). Collectively, these data reveal that GHIP serves as a novel *S. pneumoniae* virulence factor when hosts are exposed to the bacteria via the intranasal infection route.

The first stage of bacterial infection involves colonizing host tissues that are in contact with the external environment. Pneumococcal infection is initiated by asymptomatic colonization of the nasopharynx, which then disseminates into the lungs and blood. Accordingly, the ability of D39 *S. pneumoniae* and its *ΔGHIP*-deficient mutant to colonize the mouse nasopharynx and disseminate into the lungs and blood were determined. The bacterial counts in the nasopharynx, lung, and blood were significantly lower in mice infected with the *ΔGHIP* strain than with the wild-type strain at early stages post-infection (12 and 24 h) (Figure 5C); in particular, the bacterial counts in the blood of *ΔGHIP*-infected mice was below the limit of detection at 12 h after infection, indicating the wild-type strains more efficiently colonized the mice than the *ΔGHIP* mutant at early phases of infection. At 36 h, the *ΔGHIP* mutant bacterial counts were still significantly reduced in the lung compared to wild-type bacteria, although the bacterial loads in the blood and nasopharynx exhibited similar levels between the strains at this time point. Collectively, these results suggest that GHIP serves as an important factor involved in colonizing the lung, nasopharynx, and blood.

### GHIP Participates in Bacterial Invasion of Host Cells

**Figure 6:**
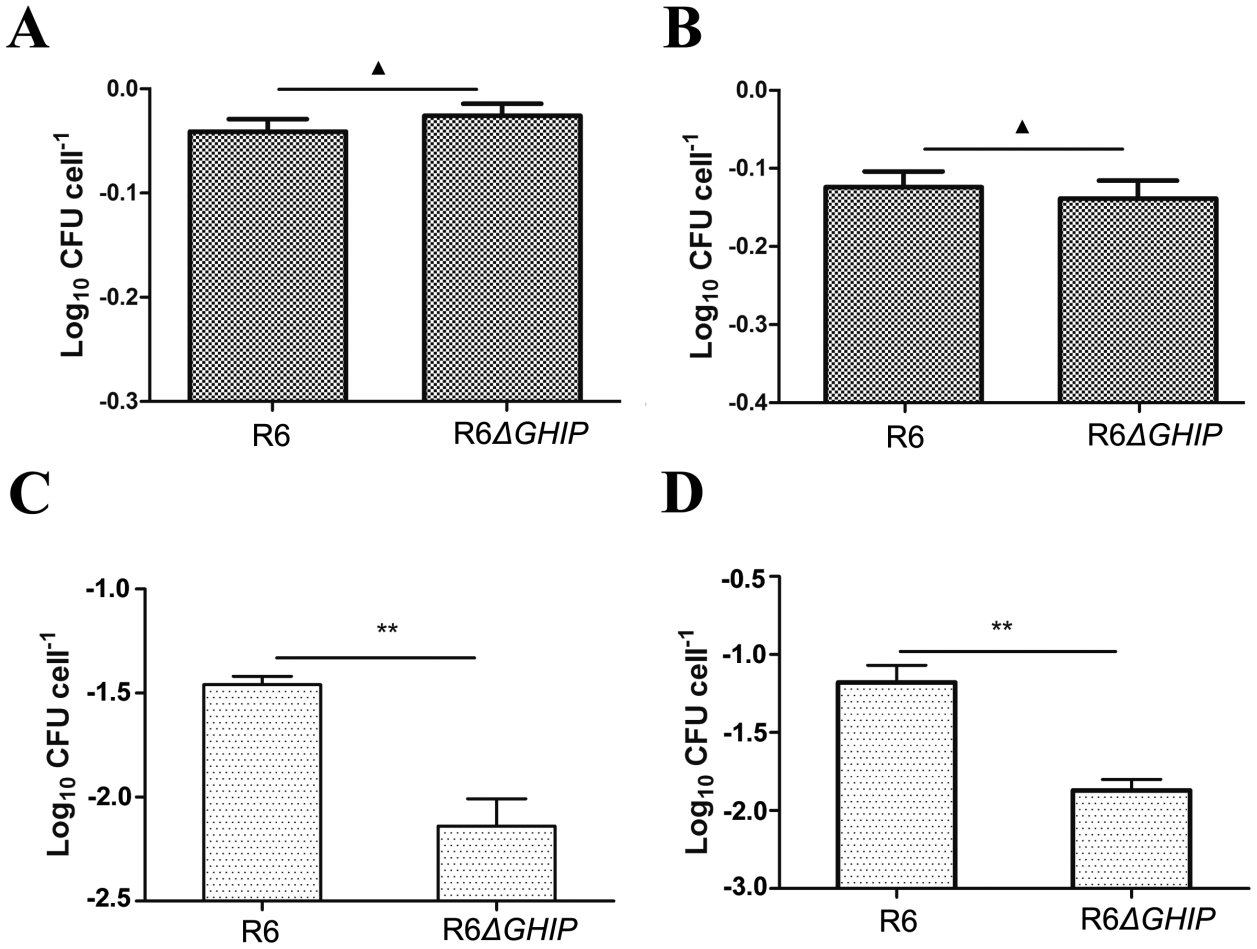
Adherence and invasion of the *ΔGHIP* mutant into host cells *in vitro*. (A) & (B) *S. pneumoniae* type R6 and their isogenic R6Δ*GHIP* mutants were examined for adherence to A549 and CNE2 cells, respectively. (C) & (D) *S. pneumoniae* type R6 and their isogenic R6Δ*GHIP* were examined for invasion into A549 and CNE2 cells, respectively. Infection experiments were conducted for 4 h at 37°C. Wild-type *S. pneumoniae* and GHIP-deficient mutants were generated to be devoid of pneumolysin in order to avoid destruction of the monolayer in the *in vitro* infection system. Scoring the number of adherent and invasive bacteria indicate that adhesion and invasion are substantially reduced for GHIP-deficient *S. pneumoniae*. Results are presented as the mean ± standard deviation for at least three independent experiments. Asterisks and triangles denote values significantly different from wild-type by Student’s *t*-test (**, P<0.01; ▴, P<0.05).

Adherence and invasion into host cells are thought to be key events in bacterial infection. To assess whether this *GHIP* gene was specifically involved in these processes, we performed precise, in-frame allelic replacement of GHIP in the unencapsulated *S. pneumoniae* R6 strains to generate the isogenic R6Δ*GHIP* mutant. Using these strains, we performed quantitative adherence and invasion assays in host cells, including the A549 and CNE2 human epithelial cell lines [37], [38]. The R6Δ*GHIP* mutant exhibited a similar hypo-adherence phenotype to the wild-type strain on both human A549 and CNE2 cell lines (P>0.05, Figure 6A & 6B), indicating that GHIP does not inhibit *S. pneumoniae* adherence to host cells. As shown in Figure 6C and 6D, invasion of both the CNE2 and A549 cell lines by the *GHIP*-deficient strain was significantly attenuated as compared to the wild-type strain. To confirm that changes in the ability to invade host epithelial cells were due to *GHIP* deletion, a functional back mutant was generated by complementation with long flanking homology polymerase reaction carrying the entire *GHIP* gene. Indeed, reintroducing the *GHIP* gene back into the mutant restored invasion ability. Taken together, these results clearly reveal that GHIP, as a novel *S. pneumoniae* virulence factor, contributes to pneumococcal disease at the invasion stage.

## Discussion

In this study, we determined the three-dimensional structure of the novel *S. pneumonia*e virulence factor, GHIP, and further investigated its various functions. GHIP is a novel type of GH25 protein containing a single-domain catalytic module, which differs from other known GH25 that contain at least two distinct domains, including a catalytic module and a choline-binding module. BLAST analysis reveals that only streptococcus bacteria encode homologs of the single-domain GHIP protein, implying that this novel surface protein might be a species-specific protein and perform some specific functions (Figure 2). The enzyme experiments further confirmed that GHIP could hydrolyze the peptidoglycan, PNP-(GlcNAc)_5_, with reduced activity. Similar to the catalytic module of other GH25s, the active site of GHIP resides in a negatively charged groove at the mouth of its β-barrel, providing a suitable complement to positively charged peptidoglycans. Sequence conservation analysis revealed that only approximately half of the residues at the active site are conserved among the GHIP homologs and known GH25s. Additionally, mutating the four strictly conserved active-site residues led to a significant loss of enzyme activity, further confirming that the active site of GHIP is in the charged groove located at the barrel mouth. Hermoso and Porter et al. suggested that GH25 enzymes could hydrolyze peptidoglycan via a general acid/base catalytic mechanism, creating a net inversion of the anomeric center of the substrate [12], [19]. Interestingly, the spatial arrangement of the four conserved acidic residues in *S. pneumoniae* GHIP is similar to that of GH25 (Figure 3), suggesting that GHIP can hydrolyze peptidoglycan via a similar acid/base catalytic mechanism. Accordingly, Asp56 and Glu156 are suggested to serve as the key catalytic residues of GHIP, and Asp154 and Asp245 participate in low-barrier hydrogen bonds with Glu156 and Asp56, respectively (2.6 Å for Asp154 and Glu156, 2.5 Å for Asp56 and Asp245). The hydrogen-bonding interactions are proposed to allow proton trafficking and protonated-state regeneration of the catalytic residues. However, a three-dimensional model of the GHIP–substrate complex will be very useful to further clarify the precise catalytic mechanism.

GHs metabolize mammalian extracellular matrices, plant cell walls, and bacterial biofilms. Some bacterially produced GH enzymes also function as virulence factors by degrading glycosides, such as peptidoglycans, polysaccharides, and glycosaminoglycans [49]–[52]. Previous research revealed that several bacteria deficient in various *GH* genes, such as AtlE of *S. epidermidis* or p60, Ami, and Auto of *L. monocytogenes*, are less virulent than their respective wild-type strains [51]–[53]. *S. pneumoniae* GH25s, including LytA, LytB, and LytC, significantly contribute to the virulence of this bacterial pathogen. The amidase, LytA, mediates bacterial lysis and phage release and is involved in nasopharyngeal colonization [8]. LytB and LytC are also involved in nasopharyngeal colonization and aid in escaping host immunity [10]. Our experiments reveal that GHIP is not required for bacterial growth or in daughter-cell separation. In addition, animal experiments demonstrated a markedly attenuated virulence by the D39*ΔGHIP* strain in mice infected through the intranasal challenge route. Collectively, these results suggest that GHIP is a novel virulence factor that participates in the ability of *S. pneumoniae* bacteria to infect hosts via the intranasal route.

*S. pneumoniae* initially colonizes the nasopharynx and then spreads to the lung and the bloodstream, causing infectious disease, such as otitis media, pneumonia, and meningitis. Multiple steps are involved during the process of *S. pneumoniae* infection, including adhesion, tissues colonization, and intracellular invasion. In this study, *ΔGHIP* mutants exhibited a reduced capacity to colonize host tissues in mice, including nasopharynx, lung, and blood, as compared to the wild-type D39 strain. During the early period of infection, the bacterial counts of the mutant strain were much lower in these tissues than of the wild-type strain. At later periods, the *ΔGHIP* mutant strain exhibited a decreased ability to colonize lung tissue, but colonization of the nasopharynx and blood were similar between the *ΔGHIP* mutant and the wild-type strain. Collectively, these results imply that deleting GHIP retards the ability of *S. pneumoniae* to colonize the nasopharynx, which then delays subsequent colonization of the lung and bloodstream.

To date, several key *S. pneumoniae* virulence factors, including hyaluronidase, neuraminidase, PspA, and pneumolysin, change the host-pathogen balance to allow bacterial colonization to switch to bacterial invasion [16]. Hyaluronidase facilitates invasion into eukaryotic cells by degrading host connective tissues [54]. Neuraminidase, including NanA and NanB, cleave sialic acid residues from a wide variety of host molecules to help expose host receptors for pneumococcal colonization, adhesion, and invasion [55]–[57]. PspA, a specific receptor for lactoferrin, plays an essential role in enabling *S. pneumoniae* to procure iron and facilitates bacterial survival and invasion [1]. In addition, *S. pneumoniae* GH25s, including LytA, LytB, and LytC, have normal lysin activity and contribute to reducing the capacity of the bacteria to adhere to host epithelial cells [8]–[10]. In this study, the *ΔGHIP*-deficient strain did not reduce the ability of *S. pneumoniae* to adhere to either the A549 or CNE2 host epithelial cell lines, whereas it significantly impaired the capacity to invade these epithelial cells. To the best of our knowledge, *S. pneumoniae* GHIP is therefore the first GH25 enzyme that functions as a virulence factor involved in host-cell invasion.

Previous studies found that PspC promotes bacterial attachment to and invasion of host cells by interacting with Factor H, a host glucoprotein [17], [18]. In this study, GHIP exhibits little autolysin or lysin activity and relatively low hydrolytic activity toward peptidoglycan, even though it folds into a TIM barrel structure similar to other GH25s. Consequently, GHIP is assumed to directly recognize the glycosyl residue of host receptor glycoproteins (such as Factor H) to participate in *S. pneumoniae* infections. However, further experiments are needed to characterize the natural GHIP substrate and clarify the GHIP-mediated mechanisms in colonization and invasion at multiple stages of *S. pneumoniae* infection.

In conclusion, the crystal structure of the novel *S. pneumoniae* GH25 enzyme, GHIP, is elucidated here. The protein has eight β/α motifs that fold into a TIM barrel structure, which is slightly different from known structures of peptidoglycan hydrolases. GHIP can hydrolyze the peptidoglycan, PNP-(GlcNAc)_5_, with reduced activity. Animal experiments confirm that *S. pneumoniae* GHIP functions as a novel virulence factor. GHIP contributes to multiple stages of the *S. pneumoniae* infection process, including colonizing the nasopharynx and invading host epithelial cells. Further experiments are therefore required to clarify the mechanism underlying bacterial infection and may highlight this novel virulence factor, GHIP, as a candidate for vaccine development.

## Acknowledgments

The authors would like to acknowledge the X-ray facility at the Key Laboratory of Structural Biology of Chinese Academy of Science and to thank Profs. Liwen Niu and Maikun Teng, School of Life Science, the University of Science and Technology of China. We also thank Prof. Jianhua He at the Institute of Applied physics and Defeng Li, Yonglin Hu, and Ying Zhang at the Institute of Biophysics, Chinese Academy of Sciences, for assistance with the SAD data collection on beam line BL17U1 at the Shanghai Synchrotron Radiation Facility (SSRF). We are also grateful to Dr. David Worthylake in Louisiana State University Health Science Center for critically reviewing and revising this manuscript. We also thank Prof Veening JW (Newcastle University, UK) for providing us with the pAE03 and pJWV25 plasmids. The structure has been deposited in the Protein Database (PDB) under accession number 4FF5.
